# Paper-Based Oil Barrier Packaging using Lignin-Containing Cellulose Nanofibrils

**DOI:** 10.3390/molecules25061344

**Published:** 2020-03-16

**Authors:** Ali H. Tayeb, Mehdi Tajvidi, Douglas Bousfield

**Affiliations:** 1 School of Forest Resources, University of Maine, 5755 Nutting Hall, Orono, ME 04469, USA; mehdi.tajvidi@maine.edu; 2 Department of Chemical and Biomedical Engineering, University of Maine, Orono, ME 04469, USA; bousfld@maine.edu; 3 Advanced Structures and Composites Center, University of Maine, 35 Flagstaff Road, Orono, ME 04469, USA

**Keywords:** lignin-containing cellulose nanofibrils, food packaging, grease barrier properties, oxygen barrier, bio-based oil proof paper

## Abstract

Environmental and health concerns are driving the need for new materials in food packaging to replace poly- or perfluorinated compounds, aluminum layers, and petroleum-based polymers. Cellulose nanofibrils (CNF) have been shown by a number of groups to form excellent barrier layers to oxygen and grease. However, the influence of lignin-containing cellulose nanofibrils (LCNF) on film barrier properties has not been well reported. Herein, thin films (16 g/m^2^) from LCNF and CNF were formed on paper substrates through a filtration technique that should mimic the addition of material at the wet end of a paper machine. Surface, barrier and mechanical attributes of these samples were characterized. The analysis on the surface free energy and water contact angle pointed to the positive role of lignin distribution in inducing a certain degree of water repellency. The observed oxygen transmission rate (OTR) and water vapor permeability (WVP) values of LCNF-coated samples were nearly similar to those with CNF. However, the presence of lignin improved the oil proof performance; these layered designs exhibited an excellent resistance to grease (kit No. 12). The attained papers with LCNF coat were formed into bowl-like containers using metal molds and a facile oven drying protocol to evaluate their resistance to oil penetration over a longer period. The results confirmed the capability of LCNF layer in holding commercially available cooking oils with no evidence of leakage for over five months. Also, an improvement in the tensile strength and elongation at break was observed in the studied papers. Overall, the proposed packaging material possesses viable architecture and can be considered as a fully wood-based alternative for the current fluorocarbon systems.

## 1. Introduction 

Due to its biocompatibility and easy-to-recycle nature, paper has been widely used for wrapping and food packaging applications as an alternative to plastics [1]. The current growing interest in this area lies, not only in the nontoxicity and renewability, but largely in its excellent specific strengths and potential chemical modifications [2]. However, the existing capillary passages within paper fibers results in no barrier to grease or oil molecules [3], this issue needs to be addressed before extending the utilization of paper-based materials for food contact applications [4]. 

The common commercial method to obtain grease barrier properties of paper involves the use of per- or polyfluoroalkyls (PFAs). Long carbon chain versions are difficult to degrade under natural conditions and are known to have some health concerns [5,6]. In addition, these compounds and their emissions can accumulate in biological systems and pollute the environment. In comparison, the short chain analogues of PFAs are less toxic and bio-accumulative than long chain versions [7,8], but even these substances, once continuously emitted, raise some concerns in relation to human health [9]. One viable practice to induce grease resistance to the paper surface, is chemical modification in which the reacted/swollen cellulosic fibers can be partially dissolved in an aqueous system and upon drying, the dissolved part may fill and reduce the porosity within the fibrous matrix. This is somehow comparable to an adhesive binding the fibers together. Some examples of such solvents are sodium hydroxide [10], NaOH-urea [11], ionic solvents [12], inorganic salt hydrates [13], etc. However, among the adopted approaches, coating an impervious layer on the paper surface has been shown to be more successful. For instance, coating materials from alginate [14], chitosan [3], and polysaccharides [15], were suggested for the production of grease resistant paper with certain degree of success. 

A recently introduced alternative is cellulose nanofibrils (CNF) that can be used for fabricating airtight films with high barrier properties against oxygen and grease, thanks to their nanosized entity (width of 5–20 nm and length of several microns), higher surface area and effective interactions between nanofibrils [16,17,18]. As reported [19], the barrier properties of CNF films comes from the extensive hydrogen bonding between cellulose hydroxyls occurring upon an intense grinding process followed by drying [2], in a way analogous to fiber refining [20].

Most petroleum-based plastics are quite permeable to oxygen [19]. This drives the use of aluminum foils or metalized layers in a number of packaging applications such as snack food packaging or aseptic packaging, though these layers cause the product to be difficult to recycle and have environmental footprint [19]. CNF has been shown to generate good oxygen and grease barrier features [21], nevertheless; little is reported on the influence of lignin-containing cellulose nanofibrils (LCNF) when producing films with regard to barrier performance. LCNF can be produced from unbleached chemical pulps, thermo-mechanical pulps (TMP), or old corrugated containers (OCC) through inexpensive refining methods [22]. These low-cost fiber sources should be of great benefit if they give similar properties to those of CNF. Moreover, LCNF may be of particular interest in view of lignin’s higher hydrophobicity (due to nonpolar hydrocarbon and benzene groups) [23], better thermal stability [24,25], antioxidant activity [26], and UV blocking features [27]. In one study, authors generated cast films of LCNF and revealed that the uniform distribution of lignin in the films can enhance the water barrier performance [23]. Also, high lignin-containing cellulose nanofibrils were explored to reinforce and enhance the barrier properties of epoxy resins; the authors showed that the load of 20–36% LCNF to the pure epoxy can double its mechanical characteristics and effectively restrict water absorption/water vapor transmission within such a hybrid system [28]. 

In this study our goal centers on fabricating an ecofriendly, recyclable paper with high oxygen and grease barrier function for food-related applications at low cost. The production of a self-standing CNF or LCNF film has been recently reported but is expected to be a slow and energy-consuming process [29]. To address this issue, we applied thin layers of CNF and LCNF on paper via vacuum filtration, to simulate the addition of material on the paper machine wet end. In this manner, some water is removed by mechanical force rather all being dried thermally. This approach is different than coating methods such as those reported by [30], or [31], where CNF suspensions were coated on paper and thermally dried. Therefore, two types of nanocellulose (CNF/LCNF) were separately deposited on a commonly available grade of printing paper to evaluate the barrier performance of each formula. The grade of printing paper was chosen as the substrate for its availability, low price and ease of recyclability. The presence of lignin between the fibrils is expected to improve the films density, surface roughness as well as the dewatering process due to its hydrophobic nature [32].

## 2. Results 

### 2.1. Morphology Differences between CNF and LCNF

**Figure 1:**
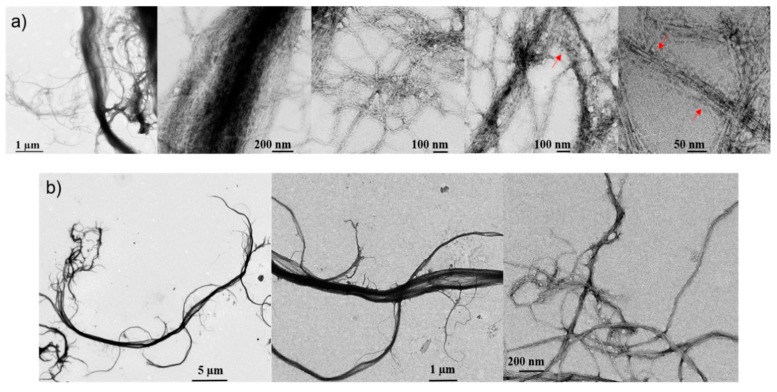
Transmission electron micrographs showing the size and morphology of nanofibrils within (**a**) LCNF and (**b**) CNF at different microscopic scales.

Understanding the morphology of the fibers is key for the fabrication of films with air-tight and grease proof qualities. The goal of this morphological study was to better apprehend the lignin distribution within the nanofibrils and its potential role to improve or damage the H-bond network in the matrix, especially once formed into a film. To answer this question, we performed both nano- and micro-scale microscopic analyses using transmission electron and optical microscopes. Figure 1 shows the TEM images of a pre-dried dilute solution of CNF and LCNF. Large micron scale fibers are evident in both samples and they have fine fibrils on the order of 50 nm. Many of the fibrils seem to be still attached to the larger fibers. For some reason, TEM images of the LCNF are clearer than those of CNF. As seen in Figure 1a, the residual lignin (roundish particles that are shown by red arrows) has surrounded the nanofibrils and prompted a configuration different than that of fibrils in CNF samples (Figure 1b). Such lignin distribution was also observed in other studies [33,34]. In theory, while high aspect ratio geometries such as fibrillar or rod-like particles are not ideal in forming tortuosity, spherical shapes may be better suited to block the diffusion [35].

**Figure 2:**
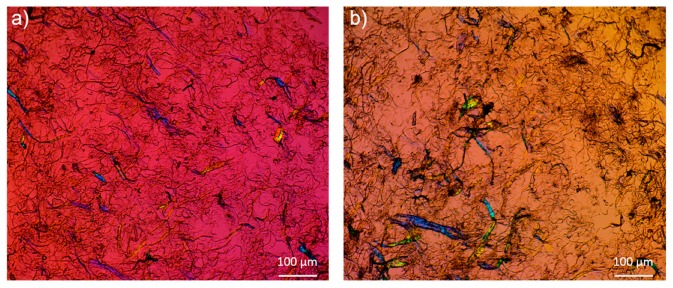
Microscopic images of nanofibrils under a polarized light. (**a**) Cellulose nanofibrils from bleached kraft, (CNF) and (**b**) cellulose nanofibrils from an unbleached kraft pulp, (LCNF).

**Figure 3:**
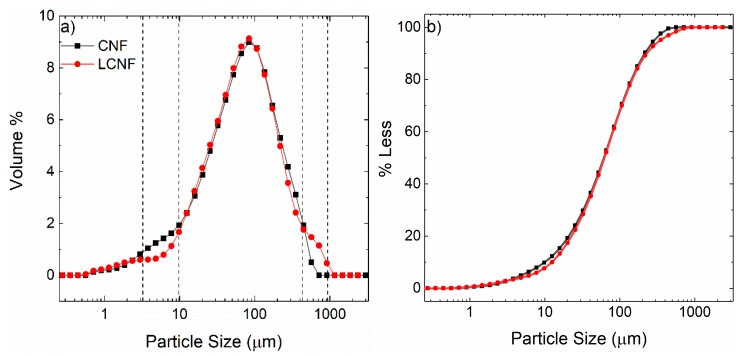
(**a**) Comparison in particle size distribution between CNF and LCNF. (**b**) The corresponding cumulative volume distribution.

Polarized optical microscopy images shed further light on the fiber morphology at a larger scale. As shown in Figure 2, the presence of lignin causes more agglomerations within the LCNF fibrils compared to the CNF case. This difference leads to a non-uniform distribution of the fibrils and variation in local density. It is possible that lignin hinders fiber liberation during the grinding process, or it influences the colloidal stability of the suspension. Similar results are noticeable in Figure 3a which displays the particle size and distribution of both samples obtained by light scattering.

Although the majority of the particles are within the range of 10–1000 µm, in agreement with our light microscopic data, a notable difference was seen in certain size ranges. CNF possessed a larger volume of smaller particles (5–10 microns) while LCNF had a higher volume of larger ones (it does not pick up the nanoscale fiber features). This inclination is more evident in Figure 3b that shows ~10% of the CNF volume is smaller than 10 µm and it does not contain any particle as large as 1000 µm.

### 2.2. Film Properties

**Figure 4:**
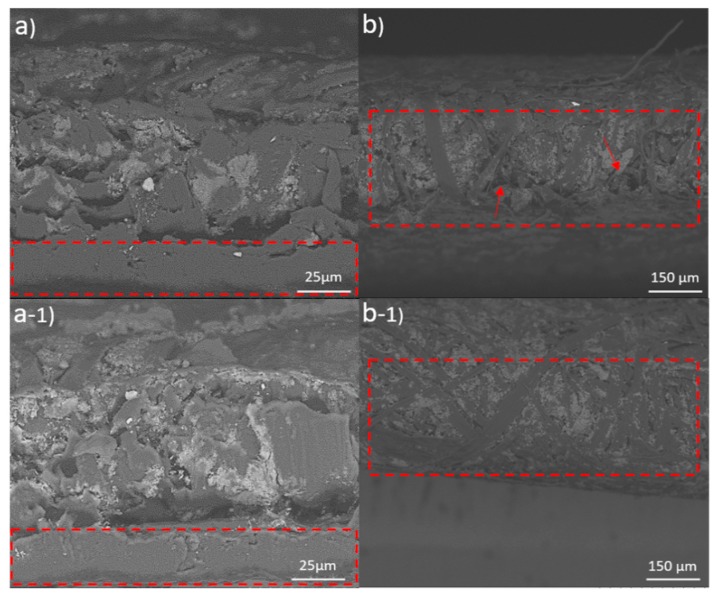
SEM images of coated papers with CNF from (**a**) cross-section and (**b**) the folded edge in tension. The corresponding LCNF images are indicated on the bottom panel as (**a-1** & **b-1**). Arrows in (**b**) show defects in the CNF film during the folding event.

No considerable difference was seen in the obtained SEM images from the cross-sections of the coated films (Figure 4a,b). The films were dense with few features at this size scale. In both cases there was a narrow gap between the paper substrate and the CNF or LCNF layer. This may be caused by shrinkage of the films during drying or it could be generated from the cutting procedure, however; the films could not be easily peeled away from the paper. Even the cross-sectional images showed no porosity of the CNF or LCNF films, though the films had large fiber fragments. Nevertheless, the analysis on the folded areas (test was performed under a 180° folding event), indicated that films with CNF coating were more susceptible to breakage compared to their LCNF counterparts. As evident in Figure 4b, the physical folding tension created several ruptures and led to the formation of some limited structural deformation at the folded regions. Arrows in Figure 4b point to the fiber separation that had occurred on the folded regions of CNF films. In the case of LCNF (Figure 4b-1), no fiber fracture from the network and/or outermost layers was observed. This can be attributed to lower brittleness that was induced by the presence of residual lignin. 

The mechanical strengths of the packaging materials is always of paramount importance [18]. The coated films improved both tensile strength and elongation at break. Generally tensile strength is mostly governed by the fiber strength as well as the interfibrillar bonding force. As seen in Figure 4a,a-1, the applied nanocellulose could partially penetrate the substrate papers and lead to higher interfiber bonds at the upper layers. This phenomenon was more obvious in CNF coated papers and thus they showed better tensile strengths.

**Figure 5:**
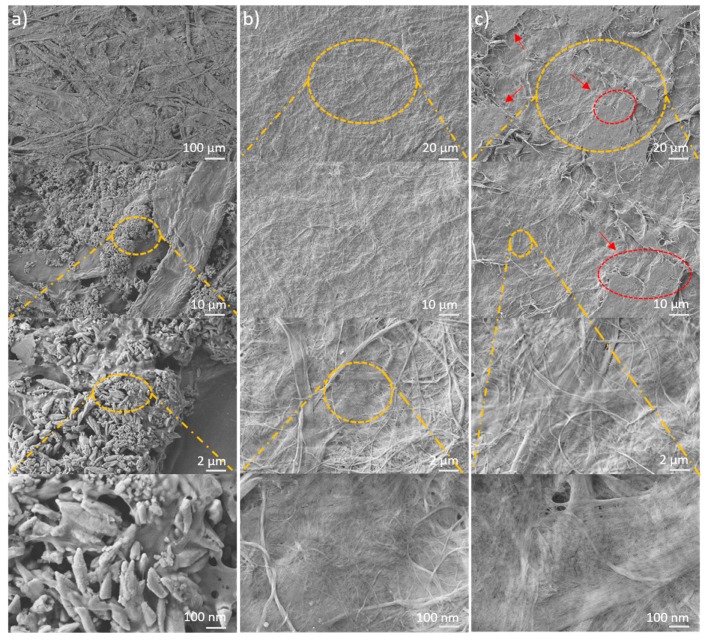
Surface SEM images of (**a**) paper substrate, (**b**) paper coated with CNF and (**c**) paper coated with LCNF at different magnitudes.

Figure 5 depicts the SEM images of paper surface before and after the coating procedure. The studied paper substrates owned a porous structure with high filler content visible on the surface (Figure 5a). However, the deposition of nanofibrils gave rise to a relatively smooth and airtight conformation on the papers’ outermost layer. No distinguishable difference in the CNF and LCNF top surface images was seen within the high-resolution images and they looked like a non-porous surface (Figure 5b,c - bottom). However, at lower resolutions certain island-like structures were detected in LCNF-coated samples that were not seen in that of CNF coats. These random assemblies are shown by red arrows and shaped into regional areas with higher uniformity (Figure 5c - top). While the exact reason is not clear, they can be originated from some sort of inconsistency due to the residual lignin. 

**Figure 6:**
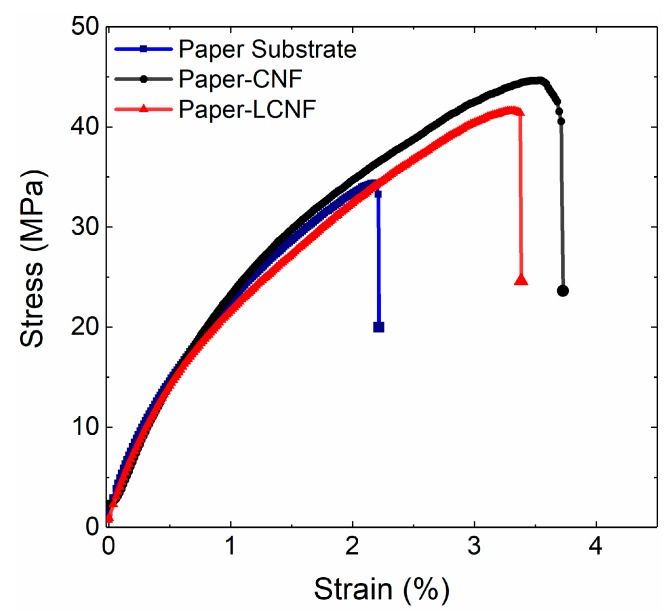
Representative stress-strain curves of the paper substrates before/after coating with CNF and LCNF.

With respect to the mechanical attributes (Figure 6), the analysis on the stress-strain behavior indicated that tensile strength increased from 36, to 41 and 45 MPa with the addition of LCNF and CNF layer respectively. The deposited layer of each formula increased the total basis weight from 78 to 94 g/m^2^; yet in the tensile data that were normalized to basis weight, papers with CNF coats showed a better strength (0.48 MPa.m^2^.g^−1^) than those with LCNF (0.44 MPa.m^2^.g^−1^) (Table 1). Also, they both led to higher elongation at break; again, the effect of CNF was more evident than LCNF. Such behavior was previously reported by our research group for LCNF produced from thermomechanical pulp [29]. Table 1 reports the average values and the standard deviations. Increases in the tensile strength of the base paper without sacrificing the strain at break is a promising observation as it implies that a thin layer of CNF or LCNF can improve the toughness as well.

One explanation for the observed difference in the mechanical attributes between the two coatings can be in part due to the role of lignin in reducing the number of effective hydrogen bonds within the cellulosic network. Similar effect has been reported for the presence of wood extractives between the cellulosic fibers [36]. Yet, the obtained average tensile values are close to some commercially available cellulose-based films such as cellulose acetate (56 MPa) [37], and cellophane (40–90 MPa) [38,39], that have been used as barrier films in food-related applications [40]. 

CNF films can form a packed network and generate excellent barrier property against oxygen [16,19]. The same theory may be applied for the LCNF films, however; there are only a few studies on the LCNF coatings’ oxygen barrier properties. The purpose of the OTR analysis in this part was to (1) evaluate whether or not the added thin layer can significantly improve the gas barrier function in the papers and (2) to compare the performance of CNF and LCNF. 

Table 2 summarizes the OTR results for both formulas. No values for the uncoated papers are presented since they were highly permeable, and the OTR tests failed. The attained outcome is quite close to our previous study on the self-standing CNF films [18] and the reported papers in the literature [41,42,43,44]. Compared to CNF, the presence of lignin increased the OTR by around 50% for the low humidity case and by 100% for the high humidity. Lignin contains organic groups that may aid in oxygen transport, just like typical polymer films that are poor barrier layers to oxygen. The results may also be explained from the decreased uniformity of the nanoscale fibrils as seen in Figure 2 that could lead to pores or defects in the LCNF films. Further, the lignin can hinder H-bonding thereby disrupting the impermeable cellulosic network.

Theoretically, lignin molecules can bestow a certain degree of hydrophobicity that should control cellulose swelling at high relative humidities but this phenomenon was not observed in either of the coated films; possibly the swelling behavior is still governed by the bulk cellulosic matrix.

The grease resistance, water barrier properties, contact angles, and surface free energies are reported in Table 3. The kit test exhibited the maximum value of “12” for both samples even after folding at complete 180^o^ fold. The surface free energy (the sum of dispersive and polar index) and the water contact angle on the coated side of the papers indicated that LCNF prompts a markedly lower surface energy and larger water contact angle values compared to CNF. In addition, the polar index was less on the LCNF surface (5.1 mN/m) than CNF (17.4 mN/m). The induced hydrophobicity did not reduce the moisture sensitivity of the film looking at the OTR values, however.

**Figure 7:**
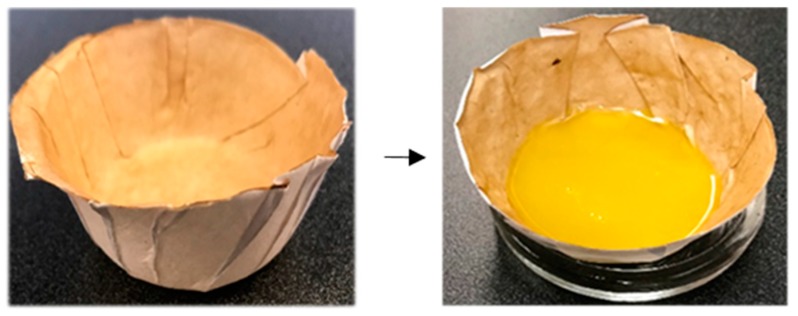
Photographs of LCNF-coated papers formed into a bowl-like shape with the capability to hold vegetable oil after five months.

Due to a tight structure, the porosity test was not feasible for the nanocellulose coated papers, however; the Gurly seconds of the uncoated paper substrate is brought in Table 3. With respect to surface smoothness, applying either type of nanocellulose led to a remarkable improvement. A lower (SU) value means a smoother surface. However, in comparison to LCNF, CNF induced a layer with an evener feature. This is in agreement with the SEM observation in Figure 5. Using a facile oven-drying method and via Teflon molds we shaped the coated papers into a bowl-like structure shown in Figure 7. These structures possessed a superb resistance to oil and could hold vegetable oil with no sign of leakage; for far more than five months. The same experiment with CNF coat on the other hand was not as successful and the bowls started to leak after a couple of hours. Although both coatings could pass the kit number 12, even under folding stress, in oil-holding experiment, LCNF was more promising, possibly for the lignin-instigated lower surface energy or better resistance to fold cracking as was evident by SEM. The role of lower surface energy in inducing oil resistance was mentioned in recent works by [1,45]. This concept can be close to low surface energy fluorocarbons that are often used within paper to confer certain level of grease proof properties.

The results of the oil-holding experiment are highly significant, given the fact that a thin layer of LCNF (16 g/m^2^) on regular paper can prompt such an excellent oil proof surface. The exact reason is not known but one hypothesis is that the lower surface energy on the LCNF surface forms a hydration layer acting as an energy barrier for the oil molecules and doesn’t allow them to penetrate the coated layer. These finding agreed with the recent study by [45] in which they reported the added layer of PVDF with lower surface energy could effectively reduce the oil-induced fouling. 

**Figure 8:**
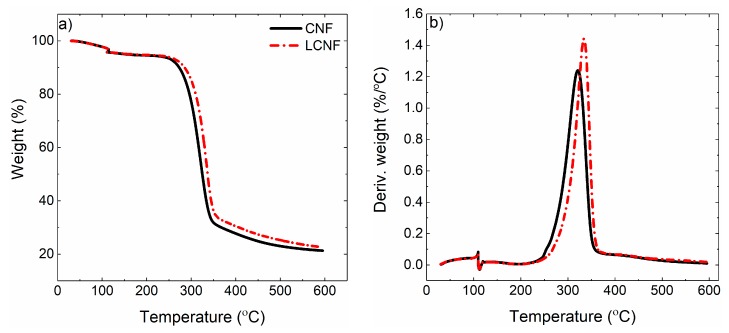
(**a**) Thermogravimetric analysis obtained from CNF and LCNF and (**b**) the corresponding derivative TG graphs.

Finally, Figure 8a,b depicts the TGA and DTG (derivative of weight loss over temperature) analysis of the pure CNF and LCNF under nitrogen atmosphere. TGA is a quantitative characterization method that measures the temperatures in which the decomposition, loss of volatile components and/or disappearance and emerging of internal bonds take place [46,47]. In certain applications grease poof papers can be in contact with hot foods or under high temperature condition, so this test was performed to evaluate the thermal stability of the coated layers for suchlike conditions. The decomposition event was seen in the range of 220–350 °C. While the onset of the degradation was quite close for both samples, the presence of lignin could delay the degradation temperature to some point. As more apparent in the DTG data, the T_max_ shifts from 321 °C (CNF) to 334 °C (LCNF) attributable to the higher thermal resistance of lignin [48].

## 3. Conclusions 

The barrier and physical properties induced by CNF and LCNF when applied on a regular paper substrate are compared. While CNF caused better oxygen barrier properties, LCNF exhibited higher grease resistance even while forming a shape. Both types penetrated the outermost interfiber network of the base paper and positively altered the elongation at break and tensile strengths, though CNF gave slightly better results. The superb oil resistance in LCNF can be ascribed to the lower polar nature and less surface energy of lignin compared to cellulose, although the actual mechanism is not fully discovered. A key result is that good properties are obtained using unbleached fibers to produce LCNF instead of using bleached fibers to produce CNF, especially when the goal is to produce a grease proof paper. Overall, our findings reveal the promising capabilities of such nanoscopic lingocellulosic entities that once engineered on the surface of paper substrate, not only can improve its physicochemical attributes but also give rise to a sustainable and fully oil-proof layer. The proposed bio-based coatings can potentially be used in food-wrapping applications where the resistance to the oil is in the center of attention. 

## 4. Experimental

### 4.1. Materials

A suspension of CNF containing 3 wt% solids had 90% fines as determined by a standard fiber sized analyzer (MorFi, Tecpap Inc., Grenoble, France); fines are the fraction of fibril bundles smaller than 200 µm in length. The CNF was obtained from the University of Maine’s Process Development Center (PDC) and was diluted to 1 wt% prior to use. The starting material for CNF was a bleached softwood kraft pulp. The solids content was examined using a moisture analyzer (Ohaus MB45 Corporation, Parsippany, NJ, USA). A board grade unbleached kraft pulp with 10–15% residual lignin (kappa number 70–100) [49], was refined similar to CNF in the same manner as a precursor to produce LCNF. The resulting LCNF possessed 90% fines and was diluted to 1% before use. The pilot-scale production of LCNF/CNF was carried out using the University of Maine’s PDC nanomaterial pilot facility. The production process mainly consisted of repulping in a hydropulper by mixing the pulp with tap water to reach a 3 wt.% consistency, pumping the resulting suspension to a buffer tank and recirculating it through a disk refiner until the desired fines content (90%) was achieved. To measure the fines content a sample of the suspension was collected every 30 min and evaluated by a TechPap MorFi analyzer. 

### 4.2. Preparation of Coated Films and Characterization

**Figure 9:**
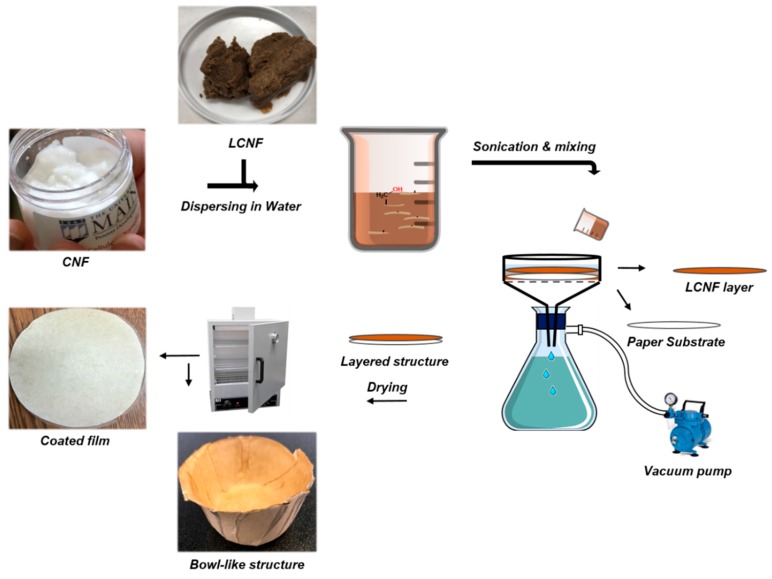
Schematic summary for fabrication process of films and oil resistant paper-based bowls via vacuum-assisted filtration coating and oven drying.

The 1 wt% CNF and LCNF slurries were sonicated for 5 min at 80% amplitude (Branson 450 Sonifier, Ultrasonics Corporation, Danbury, CT, USA) and agitated vigorously via a planetary centrifugal mixer (Thinky 310, Thinky Corporation, Tokyo, Japan) for another 5 min at 2000 rpm. Each suspension (CNF/LCNF) was further diluted and individually applied to a round cut of printing paper through vacuum-assisted filtration method. Briefly, a circular cut printing paper was placed in the bottom of the filtration funnel and the suspension of CNF or LCNF (0.1% solid content) was poured on it prior to applying the vacuum. Upon the filtration process, the wet coated structure was immediately removed and placed between two stainless steel plates under mild pressure to be oven dried at 70 °C. The process is illustrated in Figure 9. The used substrate paper was a regular printing grade (basis weight of 78 g/m^2^) with 20% ash content which was supplied by Boise Co, Boise, Idaho. The produced coated papers had a total basis weight of 94 g/m^2^ containing 16 g/m^2^ of CNF or LCNF.

Smoothness of the samples was tested via Sheffield instruments according to corresponding Tappi standard [50]. Also, the porosity values (Gurley seconds) were recorded for paper substrates via a Gurley Densometer. 

### 4.3. Surface Free Energy

The coated sides of the papers were used for the contact angle and surface free energy analysis via a Krüss mobile surface analyzer (Krüss GmbH, Hamburg, Germany). The measured contact angles were double sessile drops for both water and diiodomethane. The surface free energy (SFE) was calculated using the Owens, Wendt, Rabel, and Kaelble (OWRK) model [51].

### 4.4. Thermogravimetric Analysis (TGA)

A TGA Q500 (TA Instruments, New Castle, DE, USA) was employed for samples with both coating materials weighing between 10–20 mg to evaluate their thermogravimetric behavior under nitrogen atmosphere. The flow rate was 40 mL/min and a heating protocol from room temperature to 600 °C at 10 °C/min rate was employed. The ash content of papers was determined using a TGA Leco 701 (St. Joseph, MI, USA); 1 g of each sample was burned under air atmosphere (590 °C) and at the end the remaining ash in the crucibles was weighed. 

### 4.5. Mechanical Attributes of the Films

Tensile properties were measured by a 500 N load cell Instron testing machine (Model 5942, Instron Instruments, Norwood, MA, USA). Strips with 10 mm width were prepared for obtaining the tensile strength, strain at break as well as stress-strain curves. The cross-head speed and gauge length were set to 2 mm/min and 20 mm, respectively. At least five specimens were used, and the average values were reported.

### 4.6. Microscopic Analysis; SEM, TEM and Optical Microscopy

To compare the morphology of CNF and LCNF materials on the micron scale, we employed an AmScope light microscope (Model ME520TA, Irvine, CA, USA). The microscope was equipped with an AmScope MU900 digital camera and a halogen bulb as the light source. A bench-top TM 3000 SEM (Hitachi, Tokyo, Japan) was used to analyze the films top surface structures (an area of approximately 200 × 200 µm) before and after folding tension at various magnifications. Films were placed between two metal plates and folded (180°) for 5 min under a 5kg weight. The folded area was analyzed for crack resistance. 

Images were captured at 15 kV with no need to sputter coating the samples. ImageJ image analysis software (NIH, Bethesda, MD, USA) was employed to analyze the particle size of CNF and LCNF samples at 2000× magnification. Additionally, an NVision 40 microscope (Zeiss, Oberkochen, Germany) was utilized to obtain higher resolution surface images. The studied samples were sputter coated with Au/Pd (2.5 nm) at an accelerating voltage of 3 kV and a distance of 3.5 mm was used.

The transmission electron micrographs of nanofibrils were taken via a CM10 transmission electron microscope (TEM, Philips, Eindhoven, Netherlands) under 100 kV acceleration voltage to shed more light on the morphology differences between the used nanocelluloses as well as to provide qualitative data on distribution of residual lignin. Furthermore, the size and shape of nanofibers within cellulosic bundles were monitored. Thus, a dilute solution of nanofibrils was prepared and deposited onto an ultrathin carbon film coated on a G200 copper micro grid. The settled fibers were dried and subsequently stained by 1% uranyl acetate solution for better contrast.

### 4.7. Particle Size Distribution Analysis

A Malvern Mastersizer (Malvern Instruments, Ltd., Malvern, Worcestershire, UK) was used for analyzing the particle size/distribution of the raw materials. The principle follows the Mie theory in which the fibers are considered as “spheres” or “equivalent spheres” and via their volume, particles’ diameter is calculated. The instrument was used at 20% of feeder capacity and 4 bar of air pressure. 

### 4.8. Oil Resistance

To determine the oil resistance of the coated papers, the standard kit-test was carried out on three replicates from each formula according to the TAPPI T559 cm-12 standard [52]. Reagents with different rheology and surface energy (numbered from 1 to 12) were dropped on the films surface from a 13 mm height and wiped after 15 s by a clean cotton ball. The solution with the highest number that did not stain (darkened) the surface was considered as the passed kit number. Any denoted stain was reported as failure [52]. 

### 4.9. Water Vapor Transmission Rate

To evaluate the films’ water vapor barrier performance a gravimetric technique was applied based on the ASTM E96/E96M-16 standard [53]. Samples were cut into circles of 65 mm in diameter and upon calculating the area, they were fitted on top of the jars containing 25 mL distilled water and were tightly sealed via a silicon gasket. The coated side of the films was facing the water. The jars were left in a conditioned room (23 °C, 50% RH) and weighed before and again after 24 h. Through Equations (1) and (2), water vapor transmission rate (WVTR) and water vapor permeability (WVP) were determined:

*WVTR* = *M*/(*At*) (1)

where *M* represents the mass difference of the jar before and after the test, *A* is area, and *t* is time. Equation (2), was employed for calculating the water vapor permeability (WVP) in which the varying thickness of the films was reflected:

*WVP* = *WVTR.T*/(*P*(*sat*).Δ*RH*%) × 100 (2)

where *T* is sample thickness, *P(sat)* is the water vapor saturation pressure at 2.81 kPa–23 °C and *ΔRH* is the relative humidity difference between inside and outside of the jars. 

### 4.10. Oxygen Transmission Rate

Using the standard procedure outlined in [54], and an oxygen transmission rate test system (Ox-Tran Module 2/22, Mocon, Inc., Minneapolis, MN, USA) the oxygen transmission rate (OTR) was determined. Upon 12 min conditioning, the initial individual zero was obtained for each test at the beginning and after every two tests for 15 min. The testing cycles (each 15 min) were continued until reaching a plateau region. While exposed to 100% (O_2_) concentration, all the samples were double masked to limit the permeation area to 5.0 cm^2^. The test was carried out at 23 °C under three different humidities; 0%, 50%, and 90% RH. The same conditions were applied for both the test gas and the carrier gas. 

## Figures and Tables

**Table 1:** Films’ tensile properties (tensile strength and tensile strain) obtained from the stress-strain curves.

Coating Materials	Tensile Strength (MPa)	Tensile Strength/Basis Weight (MPa·m^2^·g^−1^)	Tensile Strain (%)
Paper (78 g/m^2^)	36 ± 3.2	0.46	2.3 ± 0.35
Coated by CNF (94 g/m^2^)	45 ± 3.8	0.48	3.5 ± 0.29
Coated by LCNF (94 g/m^2^)	41 ± 1.8	0.44	3.05 ± 0.31

**Table 2:** Oxygen transmission rates for CNF and LCNF coated films under 0, 50 and 90% relative humidity.

Sample Code	CNF			LCNF		
Relative Humidity%	0	50	90	0	50	90
OTR (cm^3^/m^2^/day)	17.4 ± 1.1	19.9 ± 0.1	171 ± 2	26.6 ± 0.5	33.7 ± 0.7	302.5 ± 2.5

**Table 3:** Grease resistance (kit number), water vapor permeability (WVP), surface free energy (SFE), smoothness and water contact angles (WCA) for the paper substrates coated with CNF and LCNF.

Characteristic	Uncoated Paper	CNF	LCNF
Kit No	0	12	12
WVP (g.mm/m^2^.kPa.day)	36.2 ± 0.5	5 ± 0.05	5.3 ± 0.1
SFE (mN/m)	-	46.3 ± 4.5	33.9 ± 4.8
Disperse (mN/m)	-	28.8 ± 2	28.7± 2.7
Polar (mN/m)	-	17.4 ± 2.5	5.1 ± 2
WCA°	-	59.4 ± 3.6	82.2 ± 4.7
Porosity (Gurley s)	14.5 ± 2.4	-	-
Smoothness (SU)	179 ± 11	140.8 ± 9	150 ± 6
Thickness (µm)	103 ± 2	120 ± 2	119 ± 3

## References

[1] Sheng J., Li J., Zhao L. (2019). Fabrication of grease resistant paper with non-fluorinated chemicals for food packaging. Cellulose.

[2] Tayeb A.H., Amini E., Ghasemi S., Tajvidi M. (2018). Cellulose Nanomaterials—Binding Properties and Applications: A Review. Molecules.

[3] Kjellgren H., Gällstedt M., Engström G., Järnström L. (2006). Barrier and surface properties of chitosan-coated greaseproof paper. Carbohydr. Polym..

[4] Tarrés Q., Oliver-Ortega H., Ferreira P.J., Àngels Pèlach M., Mutjé P., Delgado-Aguilar M. (2018). Towards a new generation of functional fiber-based packaging: cellulose nanofibers for improved barrier, mechanical and surface properties. Cellulose.

[5] Butt C.M., Muir D.C.G., Mabury S.A. (2014). Biotransformation pathways of fluorotelomer-based polyfluoroalkyl substances: A review. Environ. Toxicol. Chem..

[6] Vierke L., Staude C., Biegel-Engler A., Drost W., Schulte C. (2012). Perfluorooctanoic acid (PFOA)-main concerns and regulatory developments in Europe from an environmental point of view. Environ. Sci. Eur..

[7] Wang Z., Cousins I.T., Scheringer M., Hungerbuehler K. (2015). Hazard assessment of fluorinated alternatives to long-chain perfluoroalkyl acids (PFAAs) and their precursors: Status quo, ongoing challenges and possible solutions. Environ. Int..

[8] Holmquist H., Schellenberger S., van der Veen I., Peters G.M., Leonards P., Cousins I. (2016). Properties, performance and associated hazards of state-of-the-art durable water repellent (DWR) chemistry for textile finishing. Environ. Int..

[9] MacLeod M., Breitholtz M., Cousins I., Wit C., Persson L., Rudén C., McLachlan M. (2014). Identifying Chemicals That Are Planetary Boundary Threats. Environ. Sci. Technol..

[10] Kihlman M., Aldaeus F., Chedid F., Germgård U. (2012). Effect of various pulp properties on the solubility of cellulose in sodium hydroxide solutions. Holzforschung.

[11] Li R., Chang C., Zhou J., Zhang L., Gu W., Li C., Liu S., Kuga S. (2010). Primarily Industrialized Trial of Novel Fibers Spun from Cellulose Dope in NaOH/Urea Aqueous Solution. Ind. Eng. Chem. Res..

[12] Huber T., Pang S., Staiger M.P. (2012). All-cellulose composite laminates. Compos. Part A Appl. Sci. Manuf..

[13] Ma J., Wang Z., Zhou X., Xiao H. (2015). Self-Reinforced Grease-Resistant Sheets Produced by Paper Treatment with Zinc Chloride Solution. BioResources.

[14] Jost V., Kobsik K., Schmid M., Noller K. (2014). Influence of plasticiser on the barrier, mechanical and grease resistance properties of alginate cast films. Carbohydr. Polym..

[15] Rastogi V.K., Samyn P. (2015). Bio-based coatings for paper applications. Coatings.

[16] Tayeb A.H., Tajvidi M. (2018). Sustainable Barrier System via Self-Assembly of Colloidal Montmorillonite and Cross-linking Resins on Nanocellulose Interfaces. ACS Appl. Mater. Interfaces.

[17] Tayeb P., Tayeb A.H. (2019). Nanocellulose applications in sustainable electrochemical and piezoelectric systems: A review. Carbohydr. Polym..

[18] Zheng M., Tajvidi M., Tayeb A.H., Stark N.M. (2019). Effects of bentonite on physical, mechanical and barrier properties of cellulose nanofibril hybrid films for packaging applications. Cellulose.

[19] Wang J., Gardner D.J., Stark N.M., Bousfield D.W., Tajvidi M., Cai Z. (2018). Moisture and Oxygen Barrier Properties of Cellulose Nanomaterial-Based Films. ACS Sustain. Chem. Eng..

[20] Tayeb A.H., Latibari A.J., Tajdini A., Sepidehdam S.M.J. (2012). The influence of pulp refining on de-inking potential and strength properties of ink jet printed paper. BioResources.

[21] Hubbe M., Pruszynski P. (2020). Greaseproof Paper Products: A Review Emphasizing Ecofriendly Approaches. bioresources.

[22] Delgado-Aguilar M., González I., Tarrés Q., Pèlach M.À., Alcalà M., Mutjé P. (2016). The key role of lignin in the production of low-cost lignocellulosic nanofibres for papermaking applications. Ind. Crops Prod..

[23] Rojo E., Peresin M.S., Sampson W.W., Hoeger I.C., Vartiainen J., Laine J., Rojas O.J. (2015). Comprehensive elucidation of the effect of residual lignin on the physical, barrier, mechanical and surface properties of nanocellulose films. Green Chem..

[24] Bian H., Chen L., Dai H., Zhu J.Y. (2017). Integrated production of lignin containing cellulose nanocrystals (LCNC) and nanofibrils (LCNF) using an easily recyclable di-carboxylic acid. Carbohydr. Polym..

[25] Nair S.S., Yan N. (2015). Effect of high residual lignin on the thermal stability of nanofibrils and its enhanced mechanical performance in aqueous environments. Cellulose.

[26] Farooq M., Zou T., Riviere G., Sipponen M.H., Österberg M. (2019). Strong, Ductile, and Waterproof Cellulose Nanofibril Composite Films with Colloidal Lignin Particles. Biomacromolecules.

[27] Xing Q., Buono P., Ruch D., Dubois P., Wu L., Wang W.J. (2019). Biodegradable UV-Blocking Films through Core-Shell Lignin-Melanin Nanoparticles in Poly(butylene adipate- co-terephthalate). ACS Sustain. Chem. Eng..

[28] Nair S., Kuo P.Y., Chen H., Yan N. (2017). Investigating the effect of lignin on the mechanical, thermal, and barrier properties of cellulose nanofibril reinforced epoxy composite. Ind. Crops Prod..

[29] Horseman T., Tajvidi M., Diop C.I.K., Gardner D.J. (2017). Preparation and property assessment of neat lignocellulose nanofibrils (LCNF) and their composite films. Cellulose.

[30] Mazhari Mousavi S.M., Afra E., Tajvidi M., Bousfield D.W., Dehghani-Firouzabadi M. (2017). Cellulose nanofiber/carboxymethyl cellulose blends as an efficient coating to improve the structure and barrier properties of paperboard. Cellulose.

[31] Kumar V., Elfving A., Koivula H., Bousfield D., Toivakka M. (2016). Roll-to-Roll Processed Cellulose Nanofiber Coatings. Ind. Eng. Chem. Res..

[32] Jiang Y., Liu X., Yang Q., Song X., Qin C., Wang S., Li K. (2019). Effects of residual lignin on composition, structure and properties of mechanically defibrillated cellulose fibrils and films. Cellulose.

[33] Hong S., Song Y., Yuan Y., Lian H., Liimatainen H. (2020). Production and characterization of lignin containing nanocellulose from luffa through an acidic deep eutectic solvent treatment and systematic fractionation. Ind. Crops Prod..

[34] Huang Y., Nair S.S., Chen H., Fei B., Yan N., Feng Q. (2019). Lignin-Rich Nanocellulose Fibrils Isolated from Parenchyma Cells and Fiber Cells of Western Red Cedar Bark. ACS Sustain. Chem. Eng..

[35] Wolf C., Angellier-Coussy H., Gontard N., Doghieri F., Guillard V. (2018). How the shape of fillers affects the barrier properties of polymer/non-porous particles nanocomposites: A review. J. Memb. Sci..

[36] Tayeb A.H., Hubbe M.A., Tayeb P., Pal L., Rojas O.J. (2017). Soy Proteins As a Sustainable Solution to Strengthen Recycled Paper and Reduce Deposition of Hydrophobic Contaminants in Papermaking: A Bench and Pilot-Plant Study. ACS Sustain. Chem. Eng..

[37] Meier M.M., Kanis L.A., de Lima J.C., Pires A.T.N., Soldi V. (2004). Poly(caprolactone triol) as plasticizer agent for cellulose acetate films: influence of the preparation procedure and plasticizer content on the physico-chemical properties. Polym. Adv. Technol..

[38] Spence K.L., Venditti R.A., Rojas O.J., Habibi Y., Pawlak J.J. (2010). The effect of chemical composition on microfibrillar cellulose films from wood pulps: water interactions and physical properties for packaging applications. Cellulose.

[39] Paunonen S. (2013). Strength and barrier enhancements of cellophane and cellulose derivative films: A Review: BioResources. BioResources.

[40] Del Nobile M., Fava P., Piergiovanni L. (2002). Water transport properties of cellophane flexible films intended for food packaging applications. J. Food Eng..

[41] Aulin C., Salazar-Alvarez G., Lindström T. (2012). High strength, flexible and transparent nanofibrillated cellulose–nanoclay biohybrid films with tunable oxygen and water vapor permeability. Nanoscale.

[42] Aulin C., Gällstedt M., Lindström T. (2010). Oxygen and oil barrier properties of microfibrillated cellulose films and coatings. Cellulose.

[43] Kumar V., Bollström R., Yang A., Chen Q., Chen G., Salminen P., Bousfield D., Toivakka M. (2014). Comparison of nano- and microfibrillated cellulose films. Cellulose.

[44] Nair S., Zhu J., Deng Y., Ragauskas A. (2014). High performance green barriers based on nanocellulose. Sustain. Chem. Process..

[45] Wang Z., Lin S. (2017). The impact of low-surface-energy functional groups on oil fouling resistance in membrane distillation. J. Memb. Sci..

[46] Udoetok I.A., Dimmick R.M., Wilson L.D., Headley J.V. (2016). Adsorption properties of cross-linked cellulose-epichlorohydrin polymers in aqueous solution. Carbohydr. Polym..

[47] Pratt D.Y., Wilson L.D., Kozinski J.A., Mohart A.M. (2010). Preparation and sorption studies of β-cyclodextrin/epichlorohydrin copolymers. J. Appl. Polym. Sci..

[48] Yu J., Paterson N., Blamey J., Millan M. (2017). Cellulose, xylan and lignin interactions during pyrolysis of lignocellulosic biomass. Fuel.

[49] Smook G.A. (2002). Handbook for Pulp & Paper Technologists.

[50] TAPPI T 538 (2016). Roughness of Paper and Paperboard (Sheffield Method).

[51] Kaelble D.H. (1970). Dispersion-Polar Surface Tension Properties of Organic Solids. J. Adhes..

[52] TAPPI T 559 cm-12 (2012). Grease Resistance Test for Paper and Paperboard.

[53] Bedane A.H., Eić M., Farmahini-Farahani M., Xiao H. (2015). Water vapor transport properties of regenerated cellulose and nanofibrillated cellulose films. J. Memb. Sci..

[54] (2017). ASTM D3985-17 Standard Test Method for Oxygen Gas Transmission Rate Through Plastic Film and Sheeting Using a Coulometric Sensor.

